# Molecular and Functional Characterization of a Novel Kunitz-Type Toxin-like Peptide in the Giant Triton Snail *Charonia tritonis*

**DOI:** 10.3390/md20110686

**Published:** 2022-10-31

**Authors:** Gege Zhang, Huixia Jia, Lei Luo, Yang Zhang, Xitong Cen, Gaoyou Yao, Hua Zhang, Maoxian He, Wenguang Liu

**Affiliations:** 1 CAS Key Laboratory of Tropical Marine Bio-Resources and Ecology, Guangdong Provincial Key Laboratory of Applied Marine Biology, South China Sea Institute of Oceanology, Chinese Academy of Sciences, Guangzhou 510301, China; 2 University of Chinese Academy of Sciences, Beijing 100049, China; 3 Southern Marine Science and Engineering Guangdong Laboratory (Guangzhou), Guangzhou 511458, China; 4 Key Laboratory of Animal Models and Human Disease Mechanisms of Chinese Academy of Sciences, Kunming Institute of Zoology, Kunming 650223, China

**Keywords:** *Charonia tritonis*, salivary gland, toxin-like peptide, Kunitz domain, Ct-kunitzin

## Abstract

It has been reported that the giant triton snail (*Charonia tritonis*) inserts its large proboscis and then injects venom or acid saliva from its salivary gland into its prey, the crown-of-thorns starfish *Acanthaster planci* (COTS), paralyzing it. A full-length cDNA sequence of the *C. tritonis* Ct-kunitzin gene was obtained by RACE PCR based on a transcriptomic database constructed by our laboratory (data not published), which contains an open reading frame (ORF) sequence with a length of 384 bp including a 1–32aa Kunitz domain. The Ct-kunitzin peptide was synthesized by solid-phase polypeptide methods according to its conserved amino acid sequence, with a molecular weight of 3746.0 as well as two disulfide bonds. Renatured Ct-kunitzin was injected into mice ventricles to evaluate its potential function. Compared with the normal control group (physiological saline), the spontaneous locomotor activity of the Ct-kunitzin group decreased significantly. There was a significant effect on Ct-kunitzin on mice grip strength in the grip strength test. In addition, Ct-kunitzin exhibited remarkable biological activity in suppressing pain in the pain thresholds test. There were no significant differences between the Ct-kunitzin group and the normal control group in terms of various hematological indexes and histopathological observations. When tested in COTS, the most significant histological change was the destruction, disorganization, and significant reduction in the amount of COTS tube feet tissues. Altogether, the potential paralyzing effect on mice suggests that Ct-kunitzin is a possible agent for novel drug development.

## 1. Introduction

The giant triton snail (*Charonia tritonis*) is an echinoderm with a preference for asteroids, including crown-of-thorns starfish (COTS), which confirm its potential role in influencing COTS population biology [1,2]. Based on available information, there is evidence for a specialized venom and/or sulfuric acid in the salivary gland of *C. tritonis* that is part of the chemical arsenal used by the snail for capturing prey [3,4].

Animal venoms are comprised of different classes of proteins, peptides, and neurotransmitters, including disulfide-rich peptides [5,6]. The above chemicals show high potency and selectivity for molecular targets, including ion channels, G-protein coupled receptors, and neurotransmitter transporters. Therefore, they have potential for use as pharmacological tools and potential drugs [7,8]. To date, some venom-derived drugs have already been approved for clinical use/marketing, such as exenatide, which is an incretin mimetic from a venomous lizard, used for the treatment of type 2 diabetes mellitus. In addition, captopril is an angiotensin-converting enzyme inhibitor from snake venom that is used to treat hypertension, and ziconotide is a non-narcotic pain-relieving drug obtained from the *Conus magus* snail [9,10,11,12,13,14]. Notably, toxin-peptides from marine organisms have long been studied by biomedical researchers because of their extraordinary chemical structures and potent biological properties. Many marine venom protein/peptide-derived drugs are currently undergoing preclinical studies and clinical trials, which potentially have therapeutic potential in the treatment of chronic pain, autoimmune diseases, stroke, diabetes, and hypertension. Both of the peptides from cone snail venom, MrIA and Leconotide, have separately shown protective effects against postoperative and neuropathic pain [15,16,17,18]. Moreover, the polypeptide toxin ShK-192, isolated from sea anemones, shows excellent specificity in blocking Kv1.3 potassium channels, and is also an effective immunosuppressant [19,20].

The traditional method of purifying and separating single toxins using multi-step chromatography to study their structure and function is time-consuming and costly. With the development of high-throughput sequencing technologies, investigators can now identify and study animal toxin proteins by combining transcriptome sequencing with proteinomics technology based on mass spectrometry, and then synthesize the proteins/polypeptides by solid-phase methods, which has promise for wide applications in drug development and clinical treatments [21,22,23,24,25].

In the present study, Ct-kunitzin, a toxin peptide which shares sequence homology with Kunitz-type proteins and has two highly conserved cysteine bridges, was found. Its function was characterized by in vitro experiments. The results could provide unique insight into the design of novel drugs.

## 2. Results

### 2.1. Sequence Analysis of a Toxin-like Peptide in the Salivary Gland of C. tritonis

**Figure 1:**
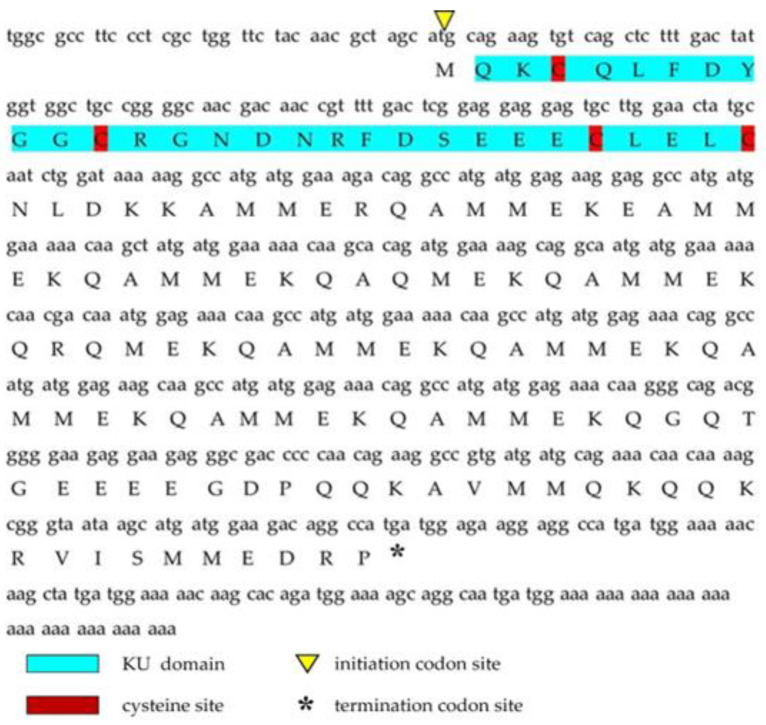
The full-length cDNA sequence of Ct-kunitzin and its deduced amino acid sequence. The KU domain is blue; the initiation codon site is the yellow upward-facing triangle; the termination codon site is the downward-facing triangle; the cysteine site is red.

**Figure 2:**
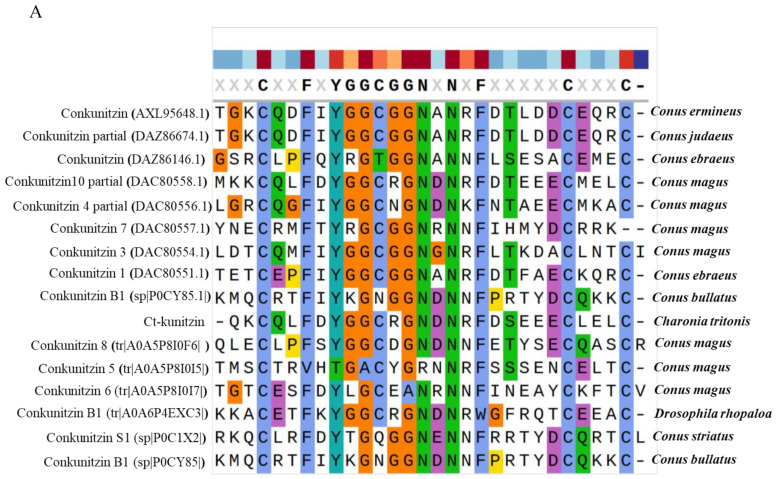

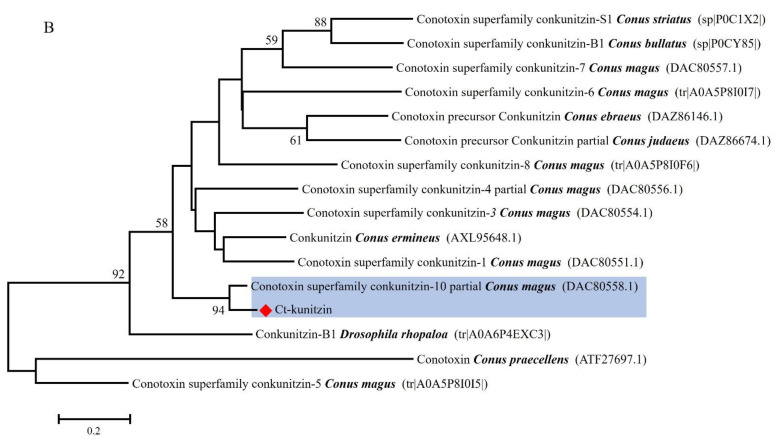
Phylogenetic analysis of venom proteins containing Kunitz domains. (**A**). Multiple alignment of Ct-kunitzin with 15 different Kunitz peptides based on the conserved sequences. The alignment was generated with the ClustalX Multiple Sequence Alignment program (version 1.83). (**B**). The Neighbor-joining (NJ) tree was constructed based on amino acid sequences using MEGA software version 6.06. The numbers below the nodes show bootstrap support values from 1000 replicates.

Analysis of next-generation sequencing of the salivary gland transcriptome of *C. tritonis* led to the identification of one transcript with high sequence similarity to conotoxin. The transcript was named Ct-kunitzin. The length of the Ct-kunitzin cDNA sequence was 458 bp and included a 127-bp ORF encoding an 81-amino acid protein (Figure 1). Ct-kunitzin was found to have a conserved domain, named KU, and was estimated to have a molecular weight of 15,195.70 kDa and a theoretical isoelectric point of 4.91 (Figure 1). Based on BLAST, we compared the conserved KU domain of Ct-kunitzin with those of different species (Figure 2A), which showed that this domain varies across species. Based on the amino acid sequences, a condensed tree was constructed by the neighbor-joining method using MEGA 6.0 (Figure 2B). This tree demonstrates that Ct-kunitzin has relatively high sequence identity with conkunitzin-10 of *Conus magus* (DAC80558.1), and that these have a common conserved element. Phylogenetic tree analysis indicates that Ct-kunitzin is evolutionarily conserved.

### 2.2. Ct-Kunitzin Expression Analysis

**Figure 3:**
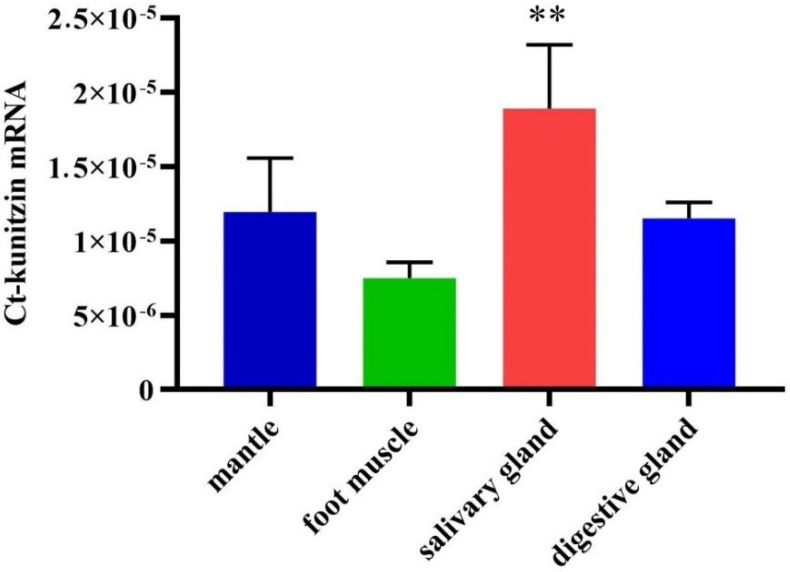
Relative mRNA expression profiles of a selected toxin-like gene from the salivary gland tissues of *C. tritonis*. A *p*-value < 0.01 was considered highly significant (**).

The tissue distribution of Ct-kunitzin was deduced from a qPCR experiment. This showed that Ct-kunitzin is highly expressed in the salivary gland of *C. tritonis* (*p* < 0.01) (Figure 3), which indicates that the salivary gland plays a toxin-secreting role in *C. tritonis*.

### 2.3. 3D Structure Determination of Ct-Kunitzin

**Figure 4:**
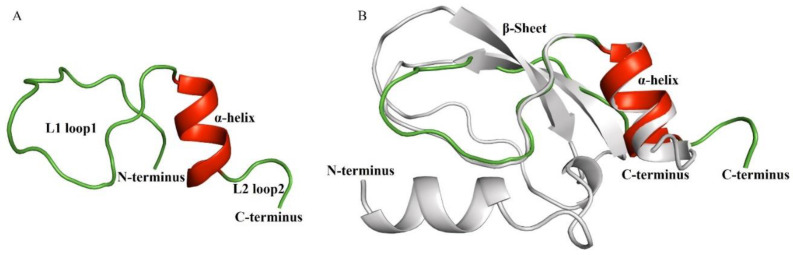
Overview of the three-dimensional structure of Ct-kunitzin and homologous structural modeling of Ct-kunitzin and a template protein named conkunitzin-10 (DAC80558.1). (**A**) 3D models of Ct-kunitzin. The main secondary structure elements are colored, with the α helix in red and loops colored in green. (**B**) Homologous modeling based on a highly homologous protein. The structures are shown in cartoon form; light grey for Ct-kunitzin, red and green for conkunitzin-10 (DAC80558.1).

Since Ct-kunitzin and conkunitzin-10 (from *Conus magus*) venom are highly similar (94% identity), a 3D model of the Ct-kunitzin peptide was created with the SWISS-MODEL based on the conkunitzin-10 structure proposed by PyMOL, highlighting the different charges of each peptide in light grey and green (Figure 4).

### 2.4. Synthesis and Renaturation of the Ct-Kunitzin Peptide

**Figure 5:**
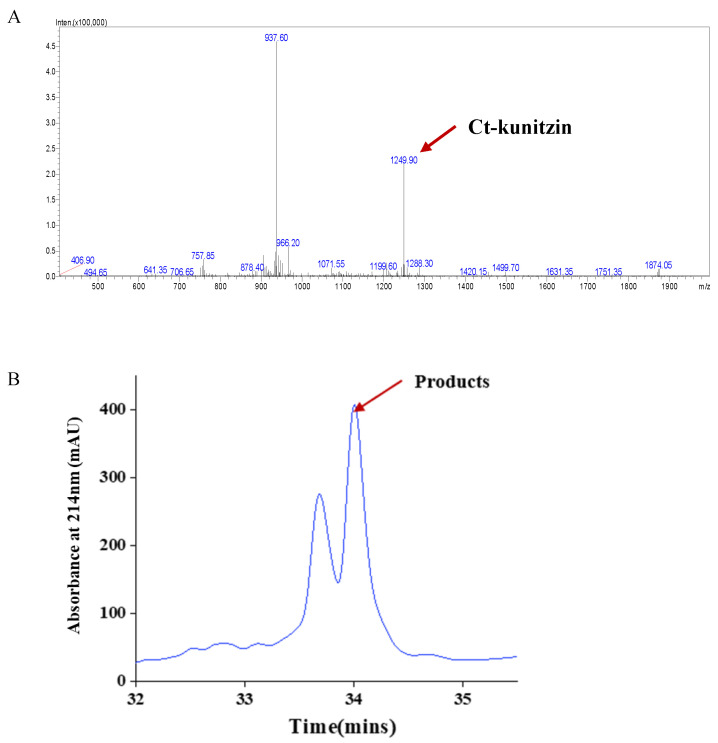
(**A**) HPLC and (**B**) mass spectra (ESI-MS) for Ct-kunitzin peptides.

The peptide had the following sequence: MQKCQLFDYGGCRGNDNRFDSEEECLELCNLD (Figure 5A). It was the defining member of a new, structurally distinct family of *Conus* peptides. The peptide was first fractionated by RP-HPLC and fractions exhibiting UV absorbance at 215 nm were selected for subsequent analysis (Figure 5B).

### 2.5. In Vitro Peptide Activities

#### 2.5.1. Effects on Motor Coordination

**Figure 6:**
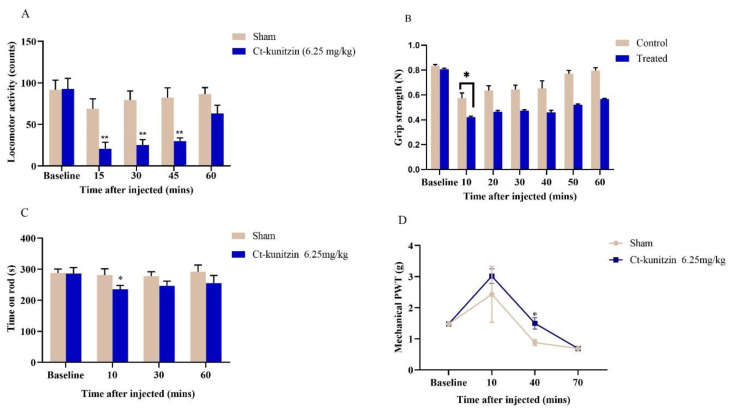
Behavioral changes after Ct-kunitzin injection in mice. (**A**). Effects of Ct-kunitzin on spontaneous locomotor activity in mice (mean ± SD, *n* = 6). (**B**). Grip strength. (**C**). Rota-rod test. (**D**). Mechanical PWT. Statistical significance values are indicated as *: *p* < 0.05, **: *p* < 0.01.

Based on the basal values measured for the experimental and control mice before administration, the difference in the frequencies of voluntary activities between the two groups was not statistically significant (*p* > 0.05), and at 15 min of administration, the frequency of voluntary activity of the experimental mice was slightly lower than that of the control group, but the difference was not significant (*p* > 0.05), presumably due to the combined effect of the anesthetic drugs used during the intracerebroventricular injection. At 30, 45, and 60 min of administration, there was a significant difference in the frequency of voluntary activity between the experimental and control groups (*p* < 0.001). At 15 min of administration, the frequency of voluntary activity of control mice reached its lowest value, and its frequency gradually recovered to near normal levels with time. The frequency of voluntary activity of experimental mice reached its lowest value at 30 min of administration, and then the frequency slowly increased with time, but at 60 min, the frequency of voluntary activity was still far below the basal value. The gripping ability of mice in the control group reached its lowest value at 10 min of drug administration, and then gradually recovered to close to the baseline value. The gripping ability of mice in the experimental group reached its lowest value at 20 min, and then showed a slow increase, but their gripping ability was still far from the baseline value at 60 min. In the control group, after injection of saline into the lateral ventricle, the rod-turning ability of the mice was slightly reduced, but it was completely restored to the normal level at 60 min. After 10 min of injection of Ct-kunitzin into the lateral ventricle, the rod-turning ability of mice in the experimental group decreased more than that in the control group, and then recovered gradually and slowly, but the rod-turning ability of mice in the experimental group was still lower than the baseline level at 60 min (Figure 6A–C).

#### 2.5.2. Effects on Pain Thresholds

The pain thresholds of both control and experimental mice were increased after ventricular injection, but the pain thresholds of the experimental mice injected with Ct-kunitzin were significantly (*p* < 0.05) greater than those of the control mice injected with saline, and their pain thresholds were still greater than those of the control mice at 40 min; but at 70 min, the pain thresholds of both experimental and control mice were smaller than the baseline values (Figure 6D).

#### 2.5.3. Effects on Blood Dynamics

**Figure 7:**
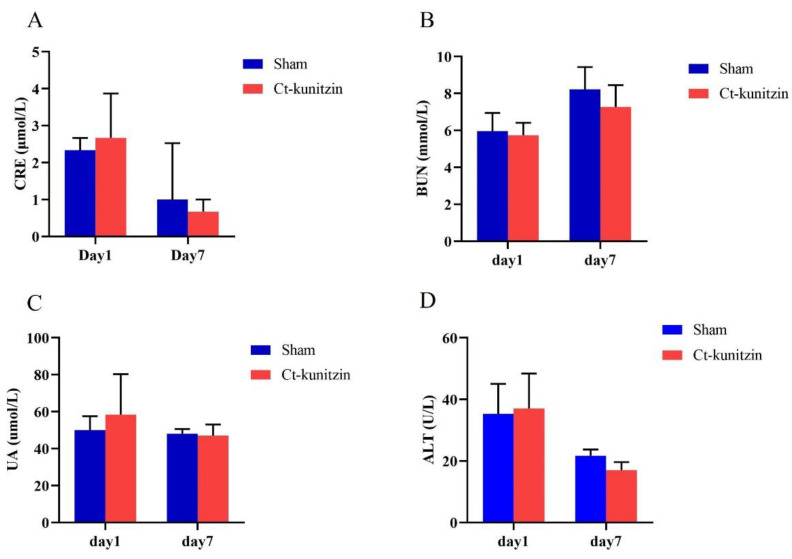
Mice plasma biochemistry on the 7th day after injection of Ct-kunitzin (mean ± SD, *n* = 6). (**A**). Serum levels of CRE. (**B**). Serum levels of BUN. (**C**). Serum levels of UA. (**D**). Serum levels of ALT.

Routine blood examinations analyzed three systems including white blood cells, red blood cells, and platelets. There were no significant differences (*p* > 0.05) between the control and experimental groups in these three indices after either 1 h or 7 d of the experiment (Table 1 and Figure 7).

#### 2.5.4. Effects on Blood Dynamics

Routine blood examinations analyzed three systems including white blood cells, red blood cells, and platelets. There were no significant differences (*p* > 0.05) between the control and experimental groups in these three indices after either 1 h or 7 d of the experiment (Figure 7).

#### 2.5.5. Effects on Vital Organs

**Figure 8:**
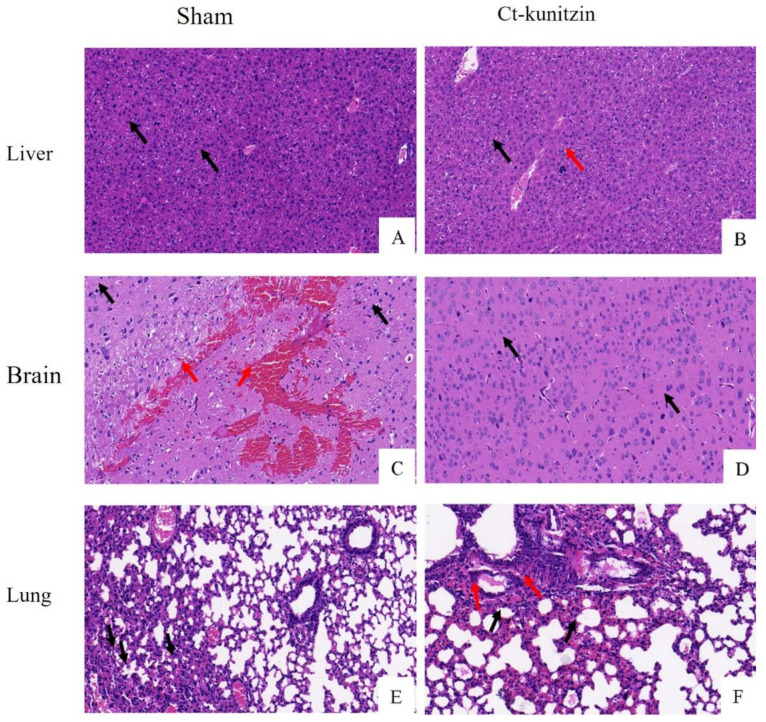
(**A**–**F**). Pathological observation of tissues in mice (H&E staining, ×200). (**A**,**C**,**E**) Control group with Sham injection. (**B**,**D**,**F**). Experimental group injected with Ct-kunitzin.

The staining results of the brain, liver, and lung tissues of control and experimental mice are shown in Figure 8, showing that when compared with the control group, Ct-kunitzin did not cause pathological changes in the brain, liver, and lung tissues of the mice. No other tissue reaction was observed, which was considered an artifact (black arrow), and no other obvious abnormalities were seen.

#### 2.5.6. Effects on COTS

**Figure 9:**
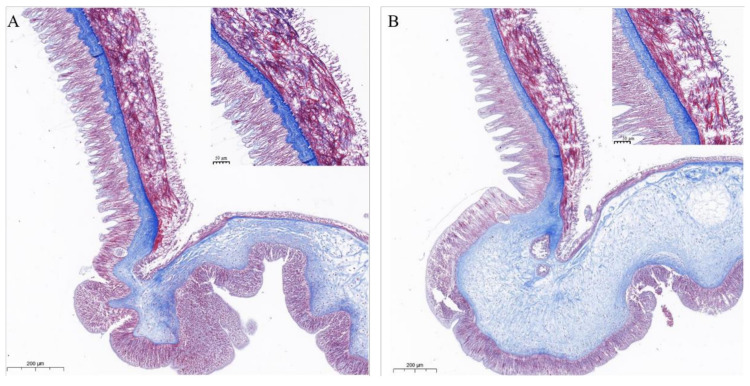
Pathological changes of tube feet tissue of COTS within 1 h of Ct-kunitzin injection. Representative histopathological pictures of the sham control stained by Masson’s Trichrome procedure. (**A**) Control group injected with saline. (**B**) Experimental group injected with Ct-kunitzin.

During the early stage of injection, COTS displayed loss of body wall turgor, dropping of spines, and production of mucus at the tip of the spines. As the injection progressed, COTS tissues showed a progressive breakdown of muscle, disorganization of connective tissue, and degeneration and disruption of the epithelium. Muscle layers continued to fragment until there was little intact muscle remaining on the collagenous connective tissues (Figure 9).

## 3. Discussion

Marine venoms are an extremely rich source of unique bioactive peptides, which have value as pharmacological tools due to their extremely high specificity and potency for particular molecular targets [26,27,28]. The Kunitz-type peptides are widely distributed in different kinds of animal venoms including those of cone snails, snakes, scorpions, etc. [29,30,31]. Previous studies have noted that the Kunitz-type peptides are good templates for potent and selective molecular probes and lead drug designs, since Kunitz peptides block potassium channels or inhibit serine proteases [32,33,34]. In this work, we identified a novel Kunitz-type toxin-like peptide from *C. tritonis*, named Ct-kunitzin. Considering that solid-phase synthetic peptide technology is very mature in the research of marine toxins like conotoxin, we also chose to chemically synthesize Ct-kunitzin [35,36,37,38,39]. As a result, compared with the conventional in vitro expression methods, the chemical method is viable for the synthesis of enough toxin peptides [40,41,42,43,44].

Based on the topology of the phylogenetic tree and molecular model, the *C. tritonis* Kunitz domain-containing peptides were evolutionarily and closely related to Conus magus-type Kunitz-like peptides, which display two highly conserved disulfide bridges. It suggested that during the course of molecular evolution, *C. tritonis* Kunitz-type toxin structures have been selected to possess neurotoxic functions [39]. Despite the disulfide bonds in Ct-kunitzin peptides not being experimentally annotated, the experimental data from the toxicity test with COTS showed that Ct-kunitzin significantly decreased the COTS swimming distance at a concentration as low as 1 mg/kg, which suggested that this *C. tritonis* Kunitz-type peptide may have muscular toxicity [41,42,43]. In parallel, Ct-kunitzin showed significant effects on the spontaneous locomotor activity of mice, but no specific damaging effect on vital organs according to plasma biochemical indices and histopathological observations. The muscular movement-related receptors, such as voltage-gated sodium (NaV), potassium (KV) [45,46,47,48], and calcium (CaV) channels and the acetylcholine receptor (AChR) are potential targets for the myotoxicity of the Ct-kunitzin peptide. Further pharmacological testing by electrophysiological techniques is required [49,50,51].

In conclusion, the putative toxins identified in this study indicated the molecular diversity of *C. tritonis* venoms and reflected conservation of core toxin families. These new results can also be used for novel protein/peptide discovery or further comparative studies to increase our understanding of the toxicology of marine venoms. From a pharmacological perspective, there has been a gradual realization that these disulfide-rich peptides might provide a greater opportunity for the development of active marine venom-derived drugs.

## 4. Materials and Methods

### 4.1. Animal Materials and Research Ethics

*C. tritonis* (N = 3) were collected from the Xisha Islands of the South China Sea and maintained in aquaria at ambient temperature (26–28 °C) and salinity (32–35‰) in Guangdong, China. Crown-of-thorns starfish (COTS) (N = 3) used in this study were collected from coastal waters in Sanya, China.

All tissue samples including salivary glands, digestive glands, and mantle and foot muscles from *C. tritonis* were removed and immediately frozen in liquid nitrogen and stored at −80 °C until isolation of the mRNA and proteins. The COTS tube feet and mice tissues (liver, lung, and brain) were removed and stored in 10% paraformaldehyde for subsequent histopathological analyses.

Four-week-old male KM mice were purchased from Guangdong Medical Laboratory Animal Center (Guangdong, China). All animal care and experiments were performed according to the guidelines and approval of the Animal Research and Ethics Committees of the Chinese Academy of Sciences (approval code: 2022-001; approval date: 25 July 2022).

### 4.2. Methods

#### 4.2.1. Identification of Transcripts Involved in Putative Toxin Gene in *C. tritonis* Salivary Gland

On the basis of existing research in the laboratory, putative toxin transcripts in the salivary gland tissue of the *C. tritonis* transcriptome were screened by searching BLASTx annotation with keywords of reference toxin-related genes reported in marine organisms. If several unique transcripts were assigned to the same reference gene, the unigene with the lowest E-value was selected as the representative.

#### 4.2.2. The 5′ and 3′ Rapid Amplification of cDNA Ends (RACE)

A cDNA fragment of 418 bp in length was chosen as the template to design outer-primer and inter-primer gene-specific primers (Supplementary Table S2) for 3′ and 5′ rapid amplification of cDNA ends (RACE). This clone represents a secondary abundant mRNA species among the six clones. The 3′ and 5′ RACE reactions were performed according to the instruction manual of the SMART RACE cDNA amplification kit (BD Bioscience Clontech, Palo Alto, CA, USA). Amplified fragments were cloned and sequenced. More than 3 independent clones of the 3′ and 5′ end of cDNAs were sequenced to eliminate possible PCR mutations.

#### 4.2.3. Nucleotide Sequence and Bioinformatics Analyses of the Putative Toxin Gene

Nucleotide sequence similarities were examined with BLAST software (https://www.ncbi.nlm.nih.gov/BLAST/, accessed on 20 August 2022). ORF prediction was performed using the ORF Finder tool (https://www.ncbi.nlm.nih.gov/orffinder/, accessed on 20 August 2022). Amino acid sequences were analyzed using the National Center for Biotechnology Information (NCBI) Conserved Domain Search (CDS) program (CDS, https://www.ncbi.nlm.nih.gov/Structure/cdd/wrpsb.cgi, accessed on 20 August 2022). The sequences with similar conserved domain architecture were predicted by the Conserved Domain Architecture retrieval Tool (CDART) program (https://www.ncbi.nlm.nih.gov/Structure/lexington/lexington.cgi?cmd=rps, accessed on 20 August 2022). Multiple sequence alignment was performed with the ClustalX program (http://www.ebi.ac.uk./clustalw) and a phylogenetic tree based on the amino acid sequences was created by using Molecular Evolutionary Genetics Analysis (MEGA) software version 6.0. The phylogenetic tree was constructed by using the neighbor-joining algorithm and performing 1000 bootstrap replications.

#### 4.2.4. qPCR Validation for Tissue Expression Profiles of Toxin-Related Genes 

The toxin-peptide gene was selected for qPCR validation. Tissues including the salivary gland, mantle, foot muscle, and digestive gland of *C. tritonis* were ground in a homogenizer (IKA, Staufen, Germany) for real-time (RT)-PCR and qPCR. Total RNA was isolated from whole tissue with TRIzol reagent (Invitrogen, Carlsbad, CA, USA), its quality checked by electrophoresis in 1.2% (*w*/*v*) agarose gel electrophoresis, and was quantitatively measured using an ultraviolet spectrophotometer (Q5000; Quawell, San Jose, CA, USA). All RNAs were treated with DNase I to avoid genomic contamination. A 1 μg sample of isolated RNA was used to synthesize first-strand cDNA using a ReverTra Ace-a first strand cDNA synthesis kit (Toyobo, Tokyo, Japan). The qPCR used a LightCycler 480 RT-PCR system (Roche, Basel, Switzerland) using SYBR (R) Premix Ex Taq™ (Toyobo) according to the manufacturer’s protocol. After amplification, fluorescent data were converted to threshold cycle (Ct) values. Concentrations of templates in samples were determined by relating Ct values to standard curves. Target gene transcript levels were normalized against reference gene transcript levels. The reference used an 18 s gene. 

#### 4.2.5. Structural Model Visualization of Ct-Kunitzin

The spatial three-dimensional structural model of Ct-kunitzin were generated using PyMOL (https://github.com/schrodinger/pymol-open-source, accessed on 26 August 2022). The other kunitzin peptide from Conus ermineus was used as a template.

#### 4.2.6. Peptide Synthesis and Renaturation

The toxin peptide (MQKCQLFDYGGCRGNDNRFDSEEECLELCNLD) was synthesized by solid-phase polypeptide synthesis according to previously reported methods [52,53]. Ct-kunitzin (10 mg) was dissolved in buffer containing (5 mM, GSH; 0.5 Mm GSSH; 0.1 M Tris-HCL; 0.1 M Nacl; pH = 9.0; 25 °C) for 24 h. Then the semipreparative high-performance liquid chromatography (HPLC) was performed on the Hitachi Primaide with a DAD detector, using an ODS column (YMC-pack ODS-A, 10 × 250 mm, 5 μm). On a C18 column using linear gradients of buffer A (0.1% trifluoroacetic acid; TFA; water) and buffer B (0.1% trifluoroacetic acid; TFA; acetonitrile). The mobile phase used a gradient of 5–50% buffer B in 45 min. The flow rate was 1 ml/min and the absorbance was monitored at 215 nm.

### 4.3. In Vitro Peptide Activities

#### 4.3.1. Administration Route

Ct-kunitzin was administered into the lateral ventricle, and the drug was administered as follows: the skin of the mouse head was grasped by the hand and stretched tightly, and the drug was administered at the intersection of the midline of the head and the anterior line of the root of the ear on both sides with a microinjector at a point 2 mm from the midline of the head. The best injection concentration of Ct-kunitzin was chosen according to a preliminary experiment (See Supplementary Table S1). A microsyringe was used to penetrate the skull and enter the ventricle, and the drug was slowly injected at a volume of 6.25 mg/kg [54].

#### 4.3.2. Animal Behaviors

Grip strength: During the grip strength test, the mice were handled by their tails and placed over the grid until all paws grasped the grid. The tail was then pulled horizontally until the mouse entirely released its hold. Three separate readings were recorded and averaged in Newtons.

Spontaneous motor activity: A multi-functional mouse autonomous activity recorder was used to record the trajectory of mice in the chamber within 5 min. The box was wiped with 75% alcohol before and after each mouse test to avoid the influence of odor on the behavior of the animals, which can lead to large errors in the experimental results [55,56].

Rota-Rod test: Mice were divided into control and test phases, and the results were used to evaluate the effects of the drug on the central nervous system of the experimental animals. From days 1−7, the mice were placed on the rotating rod fatigue apparatus for learning after 2 h of drug administration each day and were tested on day 8. The rotational speed of the rotating bar was set at 20 rpm for 10 min and the acceleration was set at 7 rpm during the learning period. The time from when the mice were placed on the rotating bar to when they fell off was recorded as the time on the bar. After the performance of each group of mice was stabilized, the test period was set to 40 rpm, and the time the mice spent on the stick was recorded [56,57,58,59].

#### 4.3.3. Pain Thresholds

The rats were stimulated vertically on the middle of the plantar area using von Frey filaments (0.07−2 g for mice and 0−6 g for rats) with different bending forces. After bending of the fiber wire, the foot was held for a period of time (about 1−3 s in mice and 3−5 s in rats), and a positive response, X, was recorded if a pain-related response occurred just after contact or within this time, and the opposite was recorded as 0. The foot lift response caused by physical activity was not recorded as a positive response. If the foot was lifted, the stimulus was changed to a fiber with less bending force, and vice versa to a fiber with more bending force. Four more values were measured after the first turning point (XO or OX) appeared, and these six data and the coefficient corresponding to the last I fiber filament were used to calculate the pain threshold using the up and down method [60,61,62,63].

#### 4.3.4. Autopsy and Histopathology

Autopsies were conducted on mice (*n* = 6) after lethal envenomation to observe gross anatomical changes such as hemorrhage, discoloration of the heart, liver, and kidney, and other abnormalities, if any, and then the tissues were fixed in 10% neutral formalin. After dehydration, the organs/tissues were incubated for 1 h in two changes of xylol. Embedding was done in paraffin wax and the blocks were sectioned at a thickness of 4–5 µm by cutting on a microtome, then stained with Harris hematoxylin for 20 min and rinsed in water for 5 min. The sections were then successively dehydrated for 2 min in 70, 80, 90, and 100% isopropanol. The processed and stained sections were then mounted permanently with DPX and examined under a light microscope [64]. The tube feet were sectioned longitudinally at a thickness of 7 µm with a Microm HM 340E microtome and the sections were collected on clean glass slides. They were stained with Masson’s trichrome or with azocarmine coupled with aniline blue and orange G [65]. The sections were then observed and photographed with a Leitz Orthoplan light microscope equipped with a DC 300F digital camera (Leica, Wetzlar, Germany).

#### 4.3.5. Blood Analysis

The crude venom at a concentration of 6.25 mg/kg body weight was administered through intracerebroventricular injection into male Kunming mice, with each weighing 18−20 g. After 1 h and at 7 days, blood samples were collected. The serum was used for enzyme and electrolytical analyses. Potassium oxalate-sodium fluoride was used as an anticoagulant for blood sugar analysis. Various hematological parameters, including total leukocyte count, total erythrocyte count, hemoglobin, hematocrit value, mean corpuscular volume, mean corpuscular hemoglobin, mean corpuscular hemoglobin concentration, and platelet count were determined using an automated cell counter (K-1000, Sysmex, Kobe, Japan). Serum glutamic-oxaloacetic transaminase, lactate dehydrogenase, and alkaline phosphatase were assayed using an autoanalyzer (Erba Smart Lab, Daman, India) by using diagnostic kits (Biocon, VoehlMarienhagen, Germany; and Raichem, San Diego, CA, USA). Blood parameters were determined using the methodologies described in the manufacturers’ respective manuals for the analytical instruments.

#### 4.3.6. Histological Procedures for COTS

Three COTS tube feet injected with Ct-kunitzin (1 mg/kg) were aseptically dissected and immediately fixed in 10% formalin. After fixation in Bouin fluid for 24 h, the tube feet tissues were dehydrated and embedded in paraffin for histology. Tissues were serially sectioned at 4 μm and stained with Masson stain. Histopathological changes of infected COTS tissues after Ct-kunitzin injection were determined using a light microscope.

## Figures and Tables

**Table 1:** Effect of injected Ct-kunitzin on hemoglobin and red blood cells.

	Sham	Ct-Kunitzin (6.25 mg/kg)	Sham	Ct-Kunitzin (6.25 mg/kg)
	Day 1		Day 7	
MCH (pg)	15.3 ± 0.6	15.9 ± 0.9	16.3 ± 1.0	16.0 ± 0.6
HGB (g·L^−1^)	115 ± 9	112 ± 6	122 ± 7	115 ± 7
MCHC (g·L^−1^)	298 ± 3	302 ± 15	302 ± 13	305 ± 6
RBC (10^12^·L^−1^)	7.5 ± 0.6	7.1 ± 0.7	7.5 ± 0.1	7.3 ± 0.6
HCT (L·L^−1^)	38.6 ± 3.2	37.2 ± 3.3	40.4 ± 0.5	37.9 ± 2.2
MCV (fL)	51.6 ± 1.9	52.8 ± 0.5	53.7 ± 1.0	52.4 ± 1.3
RDW (%)	17.0 ± 0.6	18.3 ± 0.9	16.5 ± 1.6	17.1 ± 2.1

Comparison of blood values and ratios between the two mice study groups. MCH, mean corpuscular hemoglobin; HGB, hemoglobin; MCHC, mean corpuscular hemoglobin concentration; RBC, red blood cell count; HCT, hematocrit; MCV, mean corpuscular volume; RDW, mean red blood cell distribution width.

## Data Availability

Not applicable.

## Supplementary Materials

The following supporting information can be downloaded at: https://www.mdpi.com/article/10.3390/md20110686/s1, Table S1: Effects of different doses of Ct-kunitzin on spontaneous locomotor activity in mice (Mean ± SD, *n* = 6). Table S2: Effects of different doses of Ct-kunitzin on grip strength in mice (Mean ± SD, *n* = 6). Table S3. Primers used in RACE PCR in this study.
