# Supplementary material for: Molecular and Functional Characterization of a Novel Kunitz-Type Toxin-like Peptide in the Giant Triton Snail Charonia tritonis
Source: Mar Drugs. 2022 Oct 31;20(11):686. doi: 10.3390/md20110686 (PMC9695905; doi:10.3390/md20110686)
Supplement: Supplementary file 1 [file marinedrugs-20-00686-s001.zip › marinedrugs-1900247-supplementary.pdf]

Supplementary Table S1. Effects of different doses of Ct-kunitzin on spontaneous locomotor activity in mice (Mean ± SD, n = 6).

	Dose (mg/kg)	Locomotor activity (counts) Time after injected (mins)				
		0	15	30	45	60
NC	…	91.78	68.98	79.27	82.33	86.5
Ct-kunitzin	1.25	90.56	66.79	75.84	79.77	83.69
	2.25	90.49	62.75	69.61	75.57	80.76
	3.25	91.15	59.68	62.84	68.80	77.73
	4.25	89.87	41.82	56.21	59.55	69.58
	5.25	90.64	35.91	41.49	51.34	67.62
	6.25	92.94	20.78	25.23	29.93	63.20
	7.25	90.81	21	24	28	64

Supplementary Table S2. Effects of different doses of Ct-kunitzin on grip strength in mice (Mean ± SD, n = 6).

	Dose (mg/kg)	Grip strength (N) Time after injected (mins)						
		0	10	20	30	40	50	60
NC	…	0.83	0.57	0.63	0.64	0.65	0.77	0.79
Ct-kunitzin	1.25	0.82	0.57	0.62	0.63	0.62	0.73	0.78
	2.25	0.80	0.55	0.60	0.60	0.66	0.75	0.76
	3.25	0.81	0.53	0.58	0.58	0.61	0.72	0.71
	4.25	0.82	0.50	0.56	0.59	0.63	0.69	0.74
	5.25	0.80	0.48	0.52	0.55	0.60	0.69	0.72
	6.25	0.81	0.42	0.46	0.47	0.46	0.52	0.57
	7.25	0.83	0.41	0.49	0.45	0.43	0.50	0.59

Note：Font colored with blue and red are significantly different from the NC (normal control) group. Blue font means P < 0.05, Red font means P < 0.01. NC means normal control group.

Supplementary Table S3. Primers used in RACE PCR in this study.

Name	Sequences	Primers
Ct-kunitzin-GPS3	GGAGGAGGAGTGCTTGGAACTATGC	*Ct*-kunitzin 3’ outer-primer
Ct-kunitzin-NGPS3	GTGTCAGCTCTTTGACTATGGTGGC	*Ct*-kunitzin 3’ inter-primer
Ct-kunitzin-GSP5	ATAGTTCCAAGCACTCCTCCTCCGA	*Ct*-kunitzin 5’ outer-primer
Ct-kunitzin-NGSP5	GCCACCATAGTCAAAGAGCTGACAC	*Ct*-kunitzin 5’ inter-primer
UPM(Long)	CTAATACGACTCACTATAGGGCAAG	Long-common primer
	CAGTGGTATCAACGCAGAGT	
UPS(Short)	CTAATACGACTCACTATAGGGC	Short- common primer
